# Proteome Cold-Shock Response in the Extremely Acidophilic Archaeon, *Cuniculiplasma divulgatum*

**DOI:** 10.3390/microorganisms8050759

**Published:** 2020-05-19

**Authors:** Rafael Bargiela, Karin Lanthaler, Colin M. Potter, Manuel Ferrer, Alexander F. Yakunin, Bela Paizs, Peter N. Golyshin, Olga V. Golyshina

**Affiliations:** 1 School of Natural Sciences, Bangor University, Deiniol Rd, Bangor LL57 2UW, UK; f.bargiela@bangor.ac.uk (R.B.); k.lanthaler@bangor.ac.uk (K.L.); colin.potter@bangor.ac.uk (C.M.P.); a.iakounine@bangor.ac.uk (A.F.Y.); paizsxbela@gmail.com (B.P.); p.golyshin@bangor.ac.uk (P.N.G.); 2 Centre for Environmental Biotechnology, Bangor University, Deiniol Rd, Bangor LL57 2UW, UK; 3 Systems Biotechnology Group, Department of Applied Biocatalysis, CSIC—Institute of Catalysis, Marie Curie 2, 28049 Madrid, Spain; mferrer@icp.csic.es

**Keywords:** Acidophilic archaea, acidic environments, acid mine drainage systems, *Thermoplasmatales*, *Cuniculiplasma*, proteome, cold shock

## Abstract

The archaeon *Cuniculiplasma divulgatum* is ubiquitous in acidic environments with low-to-moderate temperatures. However, molecular mechanisms underlying its ability to thrive at lower temperatures remain unexplored. Using mass spectrometry (MS)-based proteomics, we analysed the effect of short-term (3 h) exposure to cold. The *C. divulgatum* genome encodes 2016 protein-coding genes, from which 819 proteins were identified in the cells grown under optimal conditions. In line with the peptidolytic lifestyle of *C. divulgatum*, its intracellular proteome revealed the abundance of proteases, ABC transporters and cytochrome C oxidase. From 747 quantifiable polypeptides, the levels of 582 proteins showed no change after the cold shock, whereas 104 proteins were upregulated suggesting that they might be contributing to cold adaptation. The highest increase in expression appeared in low-abundance (0.001–0.005 fmol%) proteins for polypeptides’ hydrolysis (metal-dependent hydrolase), oxidation of amino acids (FAD-dependent oxidoreductase), pyrimidine biosynthesis (aspartate carbamoyltransferase regulatory chain proteins), citrate cycle (2-oxoacid ferredoxin oxidoreductase) and ATP production (V type ATP synthase). Importantly, the cold shock induced a substantial increase (6% and 9%) in expression of the most-abundant proteins, thermosome beta subunit and glutamate dehydrogenase. This study has outlined potential mechanisms of environmental fitness of *Cuniculiplasma* spp. allowing them to colonise acidic settings at low/moderate temperatures.

## 1. Introduction

The recently isolated archaea *Cuniculiplasma divulgatum* belong to the order *Thermoplasmatales* (together with *Thermoplasma*, *Picrophilus*, *Ferroplasma*, *Thermogymnomonas* and *Acidiplasma*) [1]. *C. divulgatum* and related organisms were before their isolation designated as “G-plasma” and found in metagenomic sequences from acidic environments worldwide [2,3,4]. Among them, acid mine drainage (AMD) systems generating high level of pollution via production of highly acidic and heavy metal-containing waters accommodate *C. divulgatum* and other members of the family *Cuniculiplasmataceae* [4]. Metagenomic and metaproteomic studies have previously showed high relative abundances of these microorganisms in mature biofilms in AMD systems and sulfide-rich caves [5,6]. Mechanisms underlying their successful colonisation of acidic environments and their interactions with other community members have yet to be revealed, as the wet-lab studies of physiology of *C. divulgatum* are still very limited [1,7,8]. Previous works suggested that *C. divulgatum* under defined laboratory conditions may build a partnership with archaea from “*Candidatus* Micrarchaeota” taxon of ‘DPANN’ superphylum, which outlines the ecological importance of *Cuniculiplasma* as a possible host for these ubiquitous organisms [7,8,9].

Previous studies suggested that the proteolytic lifestyle represents the main *modus operandi* of archaea belonging to the order *Thermoplasmatales* and, apparently, *Cuniculiplasma*, as well [1,10]. However, despite availability of high-quality *Cuniculiplasma* genomes, no proteogenomic studies have so far been attempted to validate sequence-guided functional predictions for both strains using experimental physiology. In this context, of particular interest are molecular mechanisms allowing *C. divulgatum* to thrive in acidic environments in the range of temperatures. Our recent studies of the *C. divulgatum* strains S5 and PM4 (isolated from AMD environments of copper mines in Spain and UK, respectively) with a rather broad range of seasonal temperatures (10–40 °C) revealed detectable growth at temperatures as low as 5 °C, whereas the optimal growth temperatures were at 37 °C [1]. Furthermore, *C. divulgatum* was detected in enrichment cultures established with Svalbard (Norway) AMD samples, characterised by low temperatures (e.g., the temperature at the sampling time point was 10.5 °C in July) [4].

The following key mechanisms of cold adaptation, as reviewed earlier, have been described in archaea [11]. Amino acids composition in the in silico proteome of cold-adapted archaea exhibited a relative increase in abundance in non-charged polar amino acids, such as glutamine and threonine [12]. Furthermore, increase of tRNA flexibility and involvement of proteins important in transcription, protein transport and folding were proposed as important mechanism for cold adaptation in archaea [11]. The cold stress response of archaeal membranes is reflected by a higher abundance of unsaturated membrane lipids (diethers), by isoprenoids’ hydroxylation and reducing in the ratio of tetraethers to diethers and in the number of pentacycli [11,13]. In bacteria, that have been studied more extensively, cold adaptation is referred to, as higher copies of genes for post-translational modifications and genome plasticity elements [14]. Cold adaptation is attributed to “cold-shock proteins” (CSPs), RNA helicases, chaperones, antioxidative enzymes and proteins of cell envelope [14]. Additionally, an increase in membrane fluidity is enabled by a decrease in saturation and an increase of polar residues in lipids [14]. Short- and branched-chain fatty acids and carotenoids were also identified as being involved in cold adaptation in bacteria [13]. Furthermore, in comparison to mesophilic enzymes, bacterial psychrophilic counterparts demonstrated certain differences in structures [14]. In addition, cold adaptation in bacteria was proposed to involve changes in central metabolic pathways, e.g., by using shortened or non-central routes, for example, of glyoxylate shunt or repression of the glycolysis and TCA cycle substituted by alternative routes [15]. Moreover, the synthesis of compatible solutes and storage polymers, such as polyhydroxyalkanoates (PHAs) were shown to be advantageous for bacterial adaptation to low temperatures [15]. A multitude of cold adaptation mechanisms were established in psychrophilic oil-degrading bacterium, *Oleispira antarctica* RB-8 that included desaturation of membrane lipids; production of compatible solutes; low-temperature-induced shift in the profile of chaperonin client proteins toward enzymes for fatty acid biosynthesis; cold-active RNA degradosome and “house-cleaning” chaperones; short-circuiting the Krebs cycle; and increased content of surface-exposed negatively charged residues in most of structurally-resolved proteins, as compared to their mesophilic counterparts [16].

To provide insight into potential mechanisms underlying the ability of *Cuniculiplasma divulgatum* to occupy low temperature niches and to grow at temperatures close to the freezing point of water, we investigated the whole proteome response of *C. divulgatum* S5^T^ cells exposed to cold shock using mass spectrometry.

## 2. Materials and Methods

### 2.1. Culture Conditions

Triplicate cultures (200 mL) of *C. divulgatum* S5 (=JCM 30642^T^ =VKM B-2941^T^) were grown under optimal conditions (37 °C, modified medium 88 DSMZ, on an orbital shaker at 100 rpm) for 90 h to reach the exponential growth phase [1]. After that, half of each culture (100 mL) was transferred into other Erlenmeyer flasks and subjected to the cold shock introduced by placing of cultures in an ice-water bath (0 °C) for 3 h. During that incubation time, the remaining halves of the cultures were maintained under optimal conditions, as above. All variants were harvested by centrifugation (8000× *g* for 15 min) and washed twice in the same sterile growth medium without beef extract.

### 2.2. Protein Extraction

Protein extraction and digestion were carried out as previously described [17]. In brief, proteins were extracted by resuspending the biomass in 300–500 µL of breakage buffer containing one tablet of Roche complete-mini protease inhibitors (without EDTA) (Roche Diagnostics Ltd., West Sussex, UK) in 7 mL of 25 mM NH_4_HCO_3_ (Sigma-Aldrich, Dorset, UK).

Acid washed glass beads (150–212 µm; 70–100 US sieve) (Sigma-Aldrich, Dorset, UK) were added to the cell pellet (200 µL). The pellet was subjected to repeated rounds of bead-beating (15 bursts of 30 s with 1 min cool-down periods on wet ice in between individual bursts). The biomass was centrifuged (10 min at 4 °C, 12,000 rcf) and the supernatant was transferred to a new Eppendorf tube (Eppendorf UK, Stevenage, UK). 250 µL of fresh breakage buffer were added to the pellet, which was then resuspended by vortexing. The bottom of the extraction vial was pierced with a heated syringe needle (BD Microlance, gauge 21G or narrower) and placed on top of a fresh Eppendorf tube. The device was inserted into a 50-mL Falcon tube (Sigma-Aldrich, Dorset, UK), and the supernatant spun through at low speed (4000 rcf) for 5 min at 4 °C to separate the beads from the resulting supernatant. The protein content of the combined supernatant and flow-through was assessed using a standard Bradford assay (Sigma-Aldrich, Dorset, UK) [18]. The protein extracts were stored at −80 °C prior to subsequent digestion.

### 2.3. Protein Digest

100 µg of protein aliquots were transferred into a 0.5-mL LoBind Eppendorf tube (Eppendorf UK, Stevenage, UK), and the volume adjusted to a total of 160 µL with fresh NH_4_HCO_3_. Proteins were denatured by adding 10 µL of 1% (w/v) of RapiGest™ (Waters UK Ltd., Wilmslow, Cheshire, UK) in 25 mM NH_4_HCO_3_, followed by a 10-min incubation (80 °C; 400 rpm). The samples were reduced by adding 10 uL of freshly prepared 60-mM Dithiothreitol (DTT) (Sigma-Aldrich, Dorset, UK) in 25-mM NH_4_HCO_3_, followed by incubation at 60 °C (10 min; 400 rpm). The samples were cooled to room temperature before alkylation was initiated by adding 10 µL of freshly prepared 180mM iodoacetamide (IAM) (Sigma-Aldrich, Dorset, UK). Samples were incubated for 30 min at room temperature in the dark.

After the addition of 4.5 µL fresh NH_4_HCO_3_ to each tube, mass spectrometry grade trypsin (Promega UK, Southampton, UK) was reconstituted in 50mM acetic acid (Sigma-Aldrich, Dorset, UK). 10 µL of a 0.2-mg/mL trypsin solution was added to the reaction. Samples were incubated for 4.5 h (37 °C; 400 rpm). Another 10 µL aliquot of trypsin was added to each digestion tube, and complete digestion was carried out overnight (37 °C; 400 rpm).

Then, 5.5 µL of acetonitrile (Sigma-Aldrich, Dorset, UK) was added to each tube and RapiGest™ was precipitated by adding of 1.5 µL of trifluoroacetic acid (TFA) (Sigma-Aldrich, Dorset, UK) and incubated for a minimum 45 min (37 °C; 400 rpm). The digest was then incubated at 4 °C for a minimum of 2 h prior to 15 min of centrifugation (14,000 rpm at 4 °C). The final volume of the digest was 221.5 µL. 180 µL of the resulting supernatant was transferred into a fresh LoBind Eppendorf tube.

Digests were diluted 1:1 with glycophosphorylase B (Uniprot Accession P00489) MassPREP™ digestion-standard (Waters UK, Wilmslow, Cheshire, UK) to give a final concentration 50 fmol/uL glycophosphorylase B. 1 µL per injection of the prepared sample was used for analysis.

### 2.4. Analytical Instrumentation and Data Acquisition

The resulting spiked digests were analysed by nanoLC-HDMS^e^ using an M-Class nano-Acquity system (Waters UK, Wilmslow, Cheshire, UK) with a trap valve manager, coupled to a Synapt G2-Si mass spectrometer (Waters UK, Wilmslow, Cheshire, UK).

The sample (1.0 μL) was loaded onto the trapping column (Waters UK, Wilmslow, Cheshire, UK; NanoEase™ M/Z Symmetry C18 100Å, 5 um, 180 um × 20 mm trap column), using partial loop injection with a sample loading time of 3 min at a flow rate of 0.3 μL/min.

The sample was resolved on an analytical column (nanoEase™ M/Z Peptide BEH C18 130Å, 1.7 μm, 75 μm × 100 mm column) using a gradient of 99% A (Optima^®^ Water with 0.1% formic acid) 1% B (Optima^®^ ACN with 0.1% formic acid) (Sigma-Aldrich, Dorset, UK) to 60% A, 40% B over 90 min at a flow rate of 0.3 μL/min and then to 15% A, 85% B over 90 min. At 95 min, the conditions returned to the initial state and held for 15 min in preparation for the next injection.

The mass spectrometry (MS) data was acquired in HDMS^e^ mode, e.g., ion mobility was enabled and all ions within a specified m/z range were observed and fragmented. In essence, four signals are available from an HDMS^e^ acquisition: firstly, a low energy MS, secondly, a high energy MS/MS, the third signal contains the lockmass data for mass axis correction and the fourth relates to the ion mobility aspect of HDMS^e^.

A mass range of 50 to 2000 Da was selected in resolution mode (ToF W mode) using a positive polarity continuum acquisition. The scan time was set at 0.5 s and a ramp transfer collision energy of 15–45 eV was used for the high energy signal. The time-of-flight analyser was calibrated before running batches against the fragment ions of glufibrinopeptide and throughout the analytical run at 6 min intervals using the Waters ZSpray™ NanoLockSpray™ source with leucine enkephaline.

### 2.5. Peptide Identification and Quantification

HDMS^e^ data was processed with Progenesis QI for proteomics version 4.1 (Nonlinear Dynamics, Newcastle upon Tyne, UK). Label-free analysis and quantification was performed using an integrated Hi^3^ workflow [19], applying standard settings for HDMS^e^ peptide/protein identification.

The Progenesis Ion Accounting workflow was used, and the data was searched against the *C. divulgatum*, strain S5^T^ proteome (Uniprot proteome ID: UP000195607) including the protein sequence of glycophosphorylase B (P00489).

Peptides were identified assuming standard trypsin degradation rules with one missed cleavage allowed. The following modifications were also included: fixed carbamidomethyl (C), and variable oxidation (M), deamidation (Q), deamidation (N). The peptide mass tolerance and fragment mass tolerance were set to auto with a 4% FDR, and ion matching requirements were set to: 3, 7, 1 for fragments/peptide, fragments/protein, and peptides/protein, respectively.

Fold-change data and quantities were determined using the absolute quantification workflow within the proteomics software Progenesis QIP (Nonlinear Dynamics, Newcastle upon Tyne, UK), which is based on the Hi^3^ algorithm. Amounts of proteins quantified in this manner are expressed in “fmol” amounts and refer to the total amount quantified in 1 µL of digest.

### 2.6. Bioinformatics Analysis

Sequences for all identified proteins were fetched from Uniprot database using the url-based API. Functional annotation and classification have been developed against eggNOG Archaea database using emapper v1.0.3, arCOG database using Blastp from BLAST 2.2.31+ tools and KEGG database using BlastKOALA. All figures have been developed using R programming language.

The mass spectrometry proteomics data have been deposited to the ProteomeXchange Consortium via the PRIDE [20,21,22] partner repository with the dataset identifier PXD017828 and 10.6019/PXD017828.

## 3. Results and Discussion

### 3.1. Analysis of the Total Intracellular Proteome of C. divulgatum Cells

Based on the complete sequence of the *C. divulgatum* genome, it encodes 2016 protein-coding genes. MS analysis of the total intracellular proteome of *C. divulgatum* revealed the presence of 819 different proteins, from which 747 proteins were found to be quantifiable (e.g., fmol amounts and ratios could be calculated).

Comparison of protein abundances in *C. divulgatum* cells at control conditions and after cold shock demonstrated that the levels of 582 proteins were similar in both types of cells suggesting that they represent the “core” proteins. One hundred and four proteins were upregulated (in comparison to control conditions) under cold shock (*p* < 0.05).

All proteomic data, including polypeptides’ locus tags, accession numbers, annotations, quantity, statistical metrics and functional categories, are presented in Supplementary Tables.

### 3.2. Proteins Expressed under Optimal Growth Conditions

Cells grown under optimal conditions demonstrated expression of proteins characteristic for active metabolism. These proteins (only data with *p*-values <0.05 are discussed) include stomatin (CSP5_0371), heme/copper-type cytochrome/quinol oxidase subunit I (CSP5_1312), ATP-dependent protease family (CSP5_1287), extracellular solute-binding protein (CSP5_1649), CUT1 family ABC transporter (CSP5_0849), TrmB family transcriptional regulator (CSP5_1241), FeCT family ABC transporter substrate-binding component (CSP5_1116), and metal-dependent hydrolase of the beta-lactamase superfamily II (CSP5_0316). Moreover, proteasome subunit a (CSP5_0278), proline dehydrogenase (CSP5_0454), PePT family ABC transporter substrate-binding component (CSP5_1067), succinate dehydrogenase/fumarate reductase, subunits A and B (CSP5_0487 and CSP5_0486), dihydrolipoyl dehydrogenase (CSP5_0253), DrugE1 family ABC transporter ATPase (CSP5_0774), small metal-binding protein (CSP5_1598), transcriptional regulator_HTH domain (CSP5_0231) and UPF0179 protein (CSP5_0191), COG category G (Carbohydrate transport and metabolism) were identified. Table S1 details other proteins expressed under optimal growth conditions. Among them, respiratory proteins for molybdenum cofactor biosynthesis (CSP5_1326), sulfide:quinone oxidoreductase (CSP5_1393), succinate dehydrogenase complex subunits (CSP5_0487 and CSP5_0486), flavoprotein, subunit beta (CSP5_1655), and V-type ATP synthase subunit A (CSP5_0035) from the functional category ‘Energy production and conversion’ might be mentioned. Expressed ‘Cell cycle control, cell division and chromosome partitioning‘ category-affiliated proteins are exemplified by chromosome partition protein Smc (CSP5_0824) and cell division protein FtsZ (CSP5_1965), whose abundance reflects the active growth of cells. Active amino acid uptake and metabolism-reared polypeptides in actively growing cells were proline dehydrogenase (CSP5_0454), PepT family ABC transporter substrate-binding component (CSP5_1067) and PepT family ABC transporter ATPase (CSP5_0340), amino acid-binding ACT domain protein (CSP5_0205) and prolyl endopeptidase (CSP5_0260). Moreover, in the ‘Carbohydrate transport and metabolism’ category, the glycogen debranching enzyme (alpha-1,6-glucosidase; CSP5_1243) was expressed. Additionally, ABC-type transporters from ‘Inorganic ion transport and defence mechanisms’ category were shown to be expressed such as Fe^3+^-hydroxamate transport system (CSP5_1116), and DsrE/DsrF-like family protein (CSP5_0480). The expressed ‘Nucleotide transport and metabolism’ category-related proteins were nucleoside-triphosphatase THEP1 (CSP5_1184) and phosphoribosylaminoimidazole carboxylase (NCAIR synthetase; CSP5_0498).

In the category ‘Coenzyme transport and metabolism‘, GTP:adenosylcobinamide-phosphate guanylyltransferase (CSP5_1294), archaeal CTP-dependent riboflavin kinase (CSP5_1177) (Flavin biosynthesis II in archaea) and FMN-dependent dehydrogenase/L-lactate dehydrogenase/type II isopentenyl diphosphate isomerase (CSP5_0048) were expressed. Furthermore, proteins of category K (Transcription) were expressed, in particular, ribbon-helix-helix CopG family protein (CSP5_1325), DNA-directed RNA polymerase, subunit E’/Rpb7 (CSP5_1267), transcriptional regulator HTH domain (CSP5_0231), transcription antiterminator NusG (CSP5_0224) and AsnC family transcriptional regulator (CSP5_0811).

Interestingly, proteins from the categories ‘Post translational modification, protein turnover and chaperones‘ were expressed under standard conditions in higher numbers compared to stressed cells. These include the proteasome 20S, alpha subunit (CSP5_0278), predicted ATP-dependent serine protease (CSP5_1287), and DnaJ-class molecular chaperone with C-terminal Zn finger domain (CSP5_1163) which were revealed in proteomic pool under standard conditions from this category.

**Figure 1:**
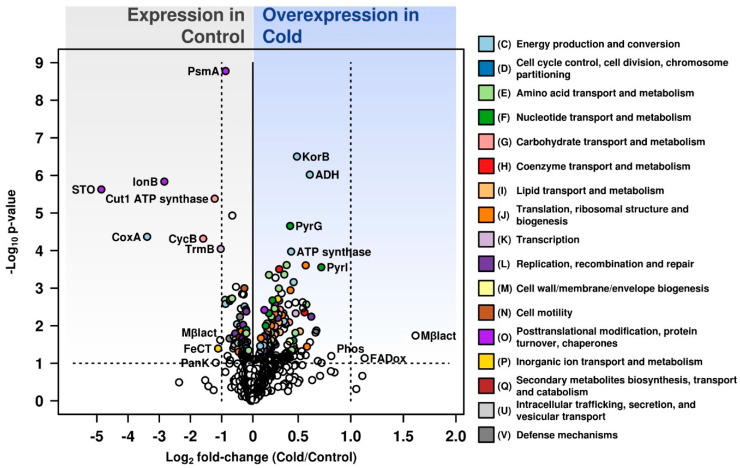
Volcano plot of normalized data for quantified proteins by HDMS^e^. Figure compares *C. divulgatum* protein biosynthesis during growth under optimal conditions (Control) and after cold shock (Cold). Data points above horizontal dashed line represent *p*-values below 0.05. Vertical dashed lines represent a two-fold change. Colour code corresponds to specific COG category assigned to identified proteins, and unassigned proteins are in the white colour. Most relevant proteins are highlighted. STO, stomatin; PsmA, proteasome alpha subunit; lonB, ATP-dependent Lon protease; Cut1-ATPase, Cut1 family ABC transporter ATPase; CoxA, cytochrome c oxidase subunit I; CycB, arabinogalactan oligomer/maltooligosaccharide transport system substrate-binding protein; TrmB, Sugar-specific transcriptional regulator TrmB; KorB, 2-oxoglutarate/2-oxoacid ferredoxin oxidoreductase subunit beta; ADH, Acryloyl-coenzyme A reductase; PyrG, Acryloyl-coenzyme A reductase; ATP synthase; Pyrl, Acryloyl-coenzyme A reductase; ElaC, metal-dependent hydrolase; FADox, FAD-dependent oxidoreductase; Mβlact, Metal-dependent hydrolase of the beta-lactamase superfamily II; PanK, Pantothenate kinase; FeCT, FeCT family ABC transporter substrate-binding component; Phos, phosphatase.

Analysis of data presented in Volcano Plot (Figure 1) revealed that under optimal growth conditions the highest expression level was shown for STO (stomatin regulator of protease activity, CSP5_0371). This protein shared high sequence similarity with related peptidases from *Acidiplasma aeolicum* (77.6% seq. identity), *Thermoplasma volcanium* (70.7%), *T. acidophilum* (69.1%) and other archaea from the order *Thermoplasmatales* without taxonomic status (e.g., *Thermoplasmatales* “E-“ and “A-plasma”). These proteolytic membrane-associated proteins are also known to occur in diverse archaea [23]. However, it must be noted that this protein has one of the lowest abundances in the proteome, only 0.001% in the cold and 0.015% under optimal growth conditions. Another minor (0.001% of the total ‘cold’ proteome) protein was expressed, the ATP-dependent protease LonB, (CSP5_1287), related with ATP-dependent proteases from *Thermoplasmatales*: *Thermoplasma acidophilum*, *T. volcanium* and *Picrophilus oshimae* with amino acid identity levels 67.61 69.93, and 70.29%, respectively. A number of archaeal LonB homologs have been characterised, including LonB from *Thermoplasma acidophilum*, which exhibited ATPase and proteolytic activity [24,25]. In *Haloverax volcanii*, LonB protease was found to control membrane lipids composition and was essential for viability [26]. Another expressed protein was PsmA, a subunit of proteasome, a large protease complex involved in protein degradation (CSP5_0278). This protein was homologous to proteasome alpha subunits from close phylogenetic relatives of *C. divulgatum, Thermoplasma* species: *T. volcanium*, and *T. acidophilum* with 76.39% and 74.25% AA sequence identity. Proteasome subunit alpha functional activity was studied in considerable details in *T. acidophilum* [27]. According to the UniProtKB, the protein belongs to the peptidase T1A family (arCOG00971) with endopeptidase activity. The expression of STO, LonB and PsmA proteins is consistent with peptidolytic lifestyle of *C. divulgatum*.

Furthermore, a Cut1 family ABC transporter ATPase (CSP5_0849) important in substrate uptake was upregulated. Proteins exhibited significant AA sequence identities with ABC transporters (ATP-binding domains) from *Picrophilus* species (*P. torridus* and *P. oshimae*, approx. 60%) and to similar proteins from acidophilic *Crenarchaeota* of *Sulfolobus*, *Acidianus* and *Acidilobus* species with 60–62% identity. A significant expression was recorded for a CoxA (CSP5_1312), heme/copper cytochrome/quinol oxidase subunit 1, which has increased its abundance from <0.001% to 0.005% of the total proteome. Interestingly, identity levels 70.22%, 54.42%, 46.08%, and 59.89%, respectively, were detected only to proteins from uncultured archaea of the order *Thermoplasmatales* namely “E-“, “A-“ and “I-plasma” and B_DKE assembly. CycB (CSP5_1649), the extracellular solute-binding/sugar ABC transporter substrate binding protein was regulated as well. Protein sequence identity of above protein with its counterpart from *Thermoplasma acidophilum* was 57.75% alongside with the ABC transporter substrate-binding proteins from thermophilic archaea of genus *Thermoproteus* (54.79%), *Vulcanisaeta* spp. (48.81–47.67%) and *Sulfolobus acidocaldarius* (57.14%). In *C. divulgatum*, this transporter could contribute to the metabolism of trehalose, a renowned compatible solute.

Furthermore, FeCT family ABC transporter substrate-binding component, Fect (CSP5_1116) with the closest identical protein from “*Ferroplasma acidarmanus*”, 47.66% and PanK, type I (CSP5_1916), are shown on Volcano plot as significantly expressed at optimal growth conditions. This protein, apart from a 62.5% sequence identity with the type I pantothenate kinase from “E-plasma”, also exhibited 53% sequence identity with bacterial type I pantothenate kinases from acidophilic bacteria *Alicyclobacillus acidocaldarius* and to a lesser extent with similar proteins from other bacilli.

Another minor protein, which was strongly expressed, FAAH fumarylacetoacetate hydrolase family protein of the MhpD superfamily (CSP5_1964), increased from 0.001 in the cold to 0.005 fmol% (Figure 1, Table S1).

### 3.3. Proteins Overexpressed under Cold Shock Conditions

The cold shock proteome of *C. divulgatum* showed a number of proteins expressed at higher levels under cold conditions (Table S1). Metal-dependent hydrolase of the beta-lactamase superfamily II (CSP5_0007, 3.06-fold) showed relatively low identity to MBL fold metallo-hydrolases proteins from *Thermoplasmatales* archaea (highest, 39.15% identity to *Thermoplasma acidophilum*). KorB; 2-oxoglutarate/2-oxoacid ferredoxin oxidoreductase subunit beta (CSP5_0285), involved in central metabolism, i.e., tricarboxylic acid cycle via the CoA-dependent oxidation of pyruvate and 2-oxoglutarate [28,29,30] was 1.4-fold overexpressed. The latter showed a high sequence identity to 2-oxoacid ferredoxin oxidoreductases from various *Thermoplasmatales* archaea. Acryloyl-coenzyme A reductase (CSP5_0778, 1.5-fold), CTP synthase (CSP5_0024, 1.34-fold), V-type ATP synthase subunit I (CSP5_0042, 1.35-fold), acetylornithine/N-succinyldiaminopimelate aminotransferase (CSP5_0018, 1.31), 50S ribosomal protein L30 (CSP5_1558, 1.48), aspartate carbamoyltransferase regulatory chain (CSP5_1108, 1.64), threonine synthase and cysteate synthase (CSP5_1396, 1.49), Cob(I)alamin adenosyltransferase (CSP5_1940, 1.47), IS3 family transposase OrfB (CSP5_1818, 1.54), ABC transporter ATPase (CSP5_0893, 1.59) and isobutyryl-CoA mutase subunit B (CSP5_0925, 1.43) were all overexpressed under cold shock conditions (Table S1). Moreover, the FADox protein, a FAD-dependent oxidoreductase of the DAO superfamily (CSP5_1916, 2-fold increase) with high sequence identity with DadA glycine/D-amino acid oxidase (deaminating)/Amino acid transport and metabolism category, showed the highest (52.35%) identity with the predicted beta subunit of SoxB-like protein (sarcosine oxidase) from uncultured “A-plasma”. Furthermore, histidine phosphatase PhoE domain protein (CSP5_0806) was 3-fold overexpressed, yet its production was at the maximal level of only 0.003% of the total proteome (Figure 1, Table S1). ADH-acryloyl coenzyme A reductase (CSP5_0778, 25% up) and D-arabinose 1-dehydrogenase from the Zn-dependent alcohol dehydrogenase family (30% up) were shown to be overexpressed. V-type ATP synthase (CSP5_0042), subunit I with 44.24% sequence identity to similar proteins from *Thermoplasmatales* archaea, showed a 27% increase in abundance. Since this enzyme complex is generating ATP, it would make perfect sense for it to be upregulated in light of increased ATP requirement for de novo protein synthesis and transport events required to meet the challenge to adapt to a new environment. The expression of this energy conservation enzyme was reported to be important for survival under stress conditions [31]. PyrL (CSP5_1108), the aspartate carbamoyltransferase regulatory subunit (Nucleotide transport and metabolism category) was detected as 50% overexpressed. The protein could be involved in allosteric regulation of aspartate carbamoyltransferase and showed 51.25% identity to similar proteins from “*Candidatus* Bathyarchaeota” archaea, from *Thermoplasma* species and *Thermoplasmatales* “E-“ and “A-plasma” variants. PyrG protein (CSP5_0024), a CTP synthase (glutamine hydrolysing) (with high levels of sequence identity to similar proteins from *Thermoplasmatales* archaea) involved in pyrimidine ribonucleotide/ribonucleoside metabolism was 20% overexpressed.

### 3.4. Differential Expression under Optimal Growth and Cold Shock Conditions of Functional Categories of Proteins

Our study has observed significant changes in expression of functional categories of proteins COG and eggnog expressed under cold shock conditions.

**Figure 2:**
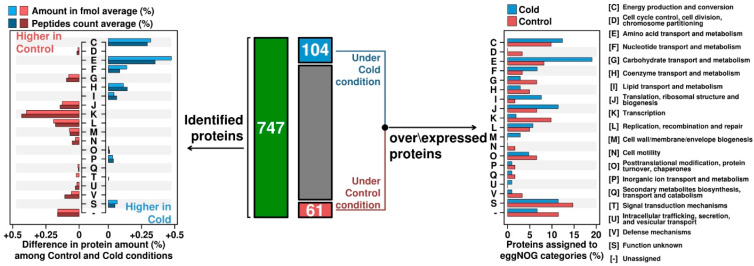
Barplots for functional assignations in eggNOG categories for identified proteins. A total number of 747 proteins have been identified and quantified by HDMS, which were after also annotated on eggNOG categories. Barplots on the left show the difference in the average of proteins abundance of all replicates (on both, fmol and peptides count) according to the functional category that have been classified. Among the 747 total proteins, 104 show upregulation (*p*-value <0.05) under the cold condition, while 61 were expressed under optimal conditions (*p*-value <0.05). Functional classification on eggNOG categories of up-regulated proteins is shown on the barplot on the right. The peptide average [%] was calculated using all injections and a percentage base on the total amount.

**Figure 3:**
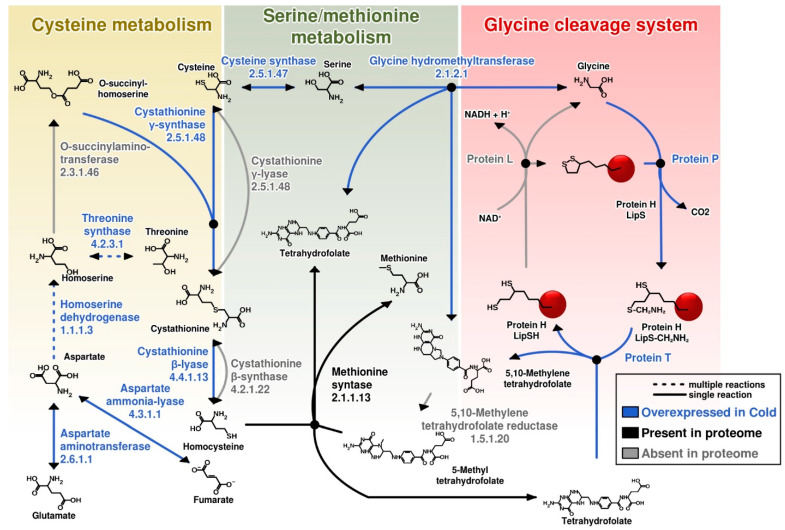
Metabolic reconstruction of amino acids biosynthesis involving overexpressed proteins under Cold condition. Most of overexpressed proteins under Cold conditions classified on *Amino acid transport and metabolism* eggNOG category (E) were related with cysteine, serine, methionine and glycine turnover. Most of the enzymes catalysing reactions on these biosynthesis pathways are overexpressed (blue arrows), some are present but not overexpressed (black arrows), and a few have not been identified on this proteomics dataset (grey arrows).

*Amino acid transport and metabolism* category showed a clear prevalence in expressed proteins in comparison to optimal growth conditions (Figure 2). Enzymes involved in aspartate, methionine and cysteine cycles were demonstrated in proteomic data to be overexpressed (cystathionine γ-synthase /cystathionine β-lyase (CSP5_0202, up 1.16 fold), threonine synthase (CSP5_1396, 1.49-fold), homoserine dehydrogenase (CSP5_0597, 1.18-fold), aspartate aminotransferase (CSP5_0812, 1.24-fold) and aspartate ammonia-lyase (CSP5_1895, 1.14-fold)). We expect that overexpression of methionine production is required for the synthesis of SAM (S-adenosylmethionine). SAM synthetase is one of the most expressed proteins and provides this important methyl-group donor, e.g., for methylation of DNA. Cysteine was another amino acid whose synthesis pathway was overexpressed under cold stress conditions. Cysteine is crucial for the disulfide bonds formation, and for the pyruvate generation. Furthermore, metabolism of both methionine and cysteine is important for production of co-factors, such as cobalamin and folic acid and for methylation of various compounds. Overexpression of proteins involved into aspartate biosynthesis might be connected to increased demand in cysteine and methionine pathways and downstream biosynthesis of ribonucleotides, purines, pyrimidines or NAD. Furthermore, the prevalence of negatively charged residues of glutamate and aspartate is characteristic for cold-active proteins and could be connected to formation of a higher number of salt bridges to increase the protein stability [32]. In addition, cysteine synthase (CSP5_0858, 1.21-fold) catalysing the bidirectional reaction between cysteine and serine was found to be overexpressed. Proteins involved into glycine cleavage system (glycine cleavage system T protein/aminomethyltransferase, CSP5_2011, 1.35-fold), glycine synthesis (glycine hydroxymethyltransferase, CSP5_0506, 1.24-fold) and lysine metabolism (CSP5_0815, succinyl-diaminopimelate desuccinylase, 1.16-fold) were detected to be overexpressed as well (Table S1, Figure 3). Additionally, glutamine (CSP5_0284, 1.38-fold) and glutamate (CSP5_0381, 1.14-fold) synthetases, 4-aminobutyrate aminotransferase (CSP5_0018, 1.31-fold), gamma-glutamyltranspeptidase (CSP5_0736, 1.29-fold), carbamate kinase (CSP5_1748, 1.18-fold) and ornithine carbamoyltransferase (CSP5_0653, 1.18-fold) were upregulated.

*Translation, including ribosome structure and biogenesis* proteins that were upregulated in cold conditions included asparagine synthetase/aspartyl/asparaginyl-tRNA synthetase (CSP5_1405, 1.34-fold), proline-tRNA ligase (CSP5_0816, 1.19), leucyl-tRNA synthetase (CSP5_1329, 1.18), phenylalanyl-tRNA synthetase subunit beta (CSP5_1324, 1.14) and alanine-tRNA ligase (CSP5_0404, 1.49-fold).

These observations imply the importance of proteins involved in amino acids metabolism, translation, and ribosomal biogenesis for cold shock resistance of *C. divulgatum* cells. The shock response to the longer (up to 5 h) exposure to the cold led to an increase in the biosynthesis of certain amino acids, as previously observed in cells of *Pyrococcus furiosus* [33]. In general, genes encoding proteins engaged in translation, transport of solutes, amino acids biosynthesis, carbon and tungsten metabolism together with some hypothetical proteins were mentioned as upregulated after early, late shock and adapted response conditions [33].

*Carbohydrate transport and metabolism* category-related proteins that were overexpressed in the proteome of cold shock-imposed cells of *C. divulgatum* included acryloyl-coenzyme A reductase alcohol dehydrogenase (CSP5_0778, 1.52-fold), glyceraldehyde-3-phosphate dehydrogenase (CSP5_0332, 1.21), ribokinase (PfkB family carbohydrate kinase) (CSP5_1289, 1.13), and short-chain dehydrogenase/reductase (CSP5_1330, 1.13). The numbers of proteins of this category that were upregulated in cold-stressed proteome exceeded those expressed under optimal growth conditions (Figure 2). All this suggests that the cold shock has induced defined metabolic changes reflected in intracellular carbohydrate metabolism. Further experimental work is required to reveal more details of this metabolic shift.

*Nucleotide transport and metabolism* category proteins expressed under cold conditions were detected to be CTP synthase (CSP5_0024, 1.34-fold), aspartate carbamoyltransferase regulatory chain (CSP5_1108, 1.64-fold), S-methyl-5’-thioadenosine phosphorylase (CSP5_0442, 1.19-fold), chlorohydrolase (CSP5_1765, 1.16-fold), phosphoribosylaminoimidazole-succinocarboxamide synthase (CSP5_1136, 1.14), adenylosuccinate synthetase (CSP5_0246, 1.37), NUDIX family hydrolase (CSP5_0459, 1.21-fold) and nucleoside diphosphate kinase (CSP5_1342, 1.36).

*Coenzyme transport and metabolism* category overexpressed proteins under cold conditions exceeded those from same category under optimal growth settings (Figure 2). In this category, following proteins were overexpressed in cold conditions: 8-amino-7-ketopelargonate synthase (CSP5_0319, 1.24), S-adenosylmethionine (SAM) synthetase (CSP5_0006) (as discussed above), von Willebrand factor type A protein (VWA) (CSP5_0782, 1.21), and Cob(I)alamin adenosyltransferase (CSP5_1940, 1.47).

**Figure 4:**
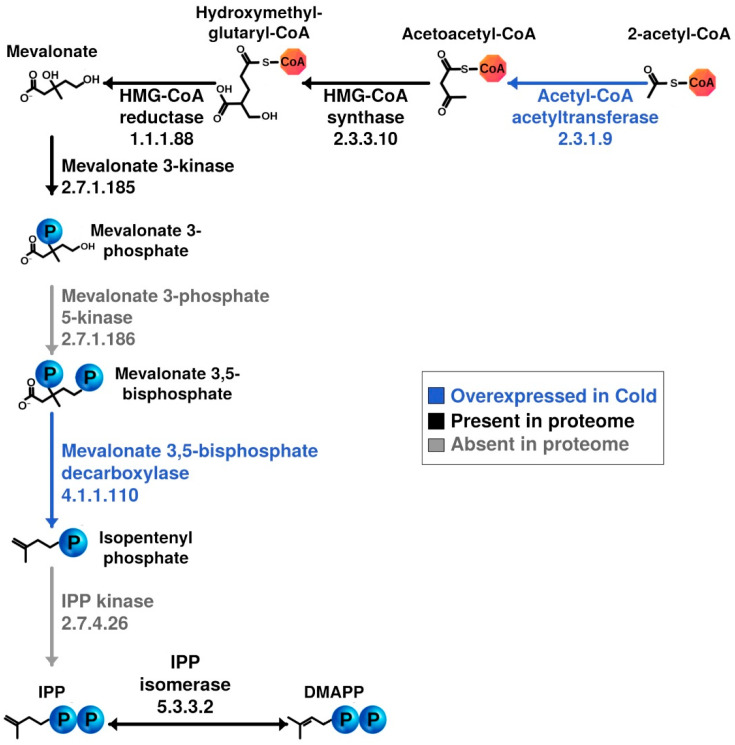
Metabolic reconstruction of membrane phospholipids biosynthesis through mevalonate pathway. Two enzymes, Acetyl-CoA acetyltransferase and Mevalonate 3,5-bisphosphate are overexpressed (blue arrows) under cold conditions, catalysing key reactions for the synthesis of mevalonate and isopentenyl bisphosphate (IPP), respectively, inside the mevalonate pathway for the synthesis of isoprenoids. These molecules are closely related with the membrane phospholipids biosynthesis, which some enzymes have been also identified, although not overexpressed, in this proteomics dataset as long as other enzymes are involved in mevalonate pathway (black arrows). Absent reactions, catalysed by enzymes not detected in this dataset, are coloured in grey.

*Lipid transport and metabolism* category included proteins of mevalonate pathway that were expressed in *C. divulgatum* (Figure 4). In particular, mevalonate 3,5-biphosphate decarboxylase, (CSP5_0962, 1.25-fold) and acetyl-CoA acetyltransferase, (CSP5_0855, 1.24-fold), were upregulated under cold shock. HMG (hydroxymethylglutaryl)-CoA synthase (CSP5_0230, 5% higher in the cold), HMG-CoA reductase (CSP5_1096, 5% lower in cold), mevalonate 3-kinase (CSP5_0479, 2.5% down in the cold) and IPP isomerase (CSP5_0048, 20% down in the cold) were found in proteomic data but were not overexpressed. However, IPP kinase (and mevalonate 3-phosphate 5-kinase) could not be identified in this dataset. Isoprenoid lipid biosynthesis from mevalonate was considered early to be associated with cold adaption in *Methanocaldococcus jannaschii* [34]. Cell membranes are known to play a vital role in stress sensing and signalling, and changes in the lipid composition of membranes have been reported in a variety of species from higher plants to archaea [35,36,37]. We detected an overexpression of two proteins involved in mevalonate pathway under cold shock conditions: acetyl-CoA-acetyltransferase and mevalonate-3,5-bisphosphate decarboxylase, the latter being important for biosynthesis of isopentenyl diphosphate, a fundamental precursor for isoprenoids [38]. We believe that efficiency of biosynthesis of isoprenoids that are the principal component of membrane tetraether lipids is important for survival strategy of *C. divulgatum*. This statement would be more solid after experimental characterisation of lipid content of cold-stressed *C. divulgatum*.

*Replication, recombination and repair* category proteins overexpressed under cold conditions were DNA topoisomerases of I (CSP5_0001, 1.12-fold) and VI (CSP5_1534, 1.12-fold) types, transposase IS3 family protein (CSP5_1818, 1.54) and others. Posttranslational modification, protein turnover and chaperones were shown to be represented by thermosome subunits alpha (CSP5_0278) and beta (CSP5_1360) (s. section ‘Most expressed proteins‘ for more details), metalloendopeptidase (CSP5_1525, 1.3-fold), protease Lon homolog (CSP5_1716, 1.13-fold), NifX family protein (CSP5_0307, 1.18-fold), and FeS assembly protein SufD of uncharacterized protein family (UPF0051), (CSP5_0664, 1.17-fold).

*Signal transduction mechanisms* category showed the presence of expressed CDC48 family ATPase of the AAA+ class (CSP5_1374, 1.21). The protein of the CDC48 family ATPase of the AAA+ class cloned from *Thermoplasma acidophilum* showed the complex appearance resembling 20S proteasome and Hsp60/GroEL [39].

*Inorganic transport and metabolism category* proteins downregulated in cold included DrsE family protein (CSP5_0480, 1.38). FeCT family ABC transporter substrate-binding component (CSP5_1116) and ATPase subunits (CSP5_1114) were detected at exactly same levels in the proteome under both conditions. However, cold shock cells of *C. divulgatum* showed the certain overexpression of rhodanese-related sulfurtransferase (CSP5_1158, 1.15-fold). Altogether, the genome of *C. divulgatum* S5 encodes four rhodanese-related sulfurtransferases and two rhodanese domains fused to Zn-dependent hydrolase of glyoxylase family. The overexpressed protein (CSP5_1158) with conserved domains on RHOD superfamily showed relatively low (36.65%) identity to metallo-beta-lactamase superfamily protein from *Thermoplasmatales* uncultured archaea of “E-plasma“ and 34.67–29.15% identity to sulfurtransferases from *Sulfobacillus* species. Interestingly, other rhodanese-related sulfurtransferases, represented in *C. divulgatum* S5 genome exhibited, along with sulfurtransferases from “A-“ and “E-plasma”) highest sequence identities with bacterial sulfurtransferases (*C. divulgatum* proteins CSP5_854, CSP5_1347, CSP5_1466, with *Acetoanaerobacterium* spp. (42.55%), and *Sulfobacillus* spp. (40.71% and 31.33%), correspondingly)). Noteworthy, CSP5_1465 and CSP5_1975 with rhodanese domain fused to Zn-dependent hydrolase of glyoxylase family were 31.3% identical to bacterial proteins MBL fold metallo-hydrolase from *Sulfobacillus* spp., from *Crenarchaeota* (45.31% identical to those from *Sulfurisphaera tokodaii* and other archaea) and from some bacteria. Consequently, we hypothesize that *Cuniculiplasma* might have experienced horizontal gene transfer of sulfurtransferases from archaeal or bacterial community members. Proteins with rhodanese-like domains were considered associated with stress in bacteria previously [40]. Functions of those proteins may include (i) chaperone activity to provide assembly of iron-sulfur complexes, (ii) detoxification of some toxic compounds (arsenate and cyanide), (iii) maintenance of redox homeostasis and (iv) biosynthesis of cofactors, enzymes and vitamins [40,41,42,43].

Altogether, the relative number from categories ‘Energy production’ and ‘Conversion and amino acid transport and metabolism’ was higher under cold conditions in comparison to optimally grown cells. In addition, ‘Nucleotide transport and metabolism‘, ‘Coenzyme transport and metabolism‘, ‘Lipid transport and metabolism‘, ‘Inorganic ion transport and metabolism‘, and proteins belonging to unknown function were overexpressed in the cold shock proteome (Figure 2, Table S1).

Comparison of our data with those in archaeon *Pyrococcus furiosus* showed certain similarities [33]. For example, the early (1–2 h) shock response in *P. furiosus* caused upregulation of proteins (17 of 55) involved in metabolism of amino acids and primary carbohydrates, in the translation and oxidoreductase-type processes and also in transport of solutes [33]. In the cold, four-fold upregulation was detected for S-adenosylmethionine synthetase in *P. furiosus*, whereas in *C. divulgatum* there was a 20% increase in this protein biosynthesis. Additionally, upregulation of oxidoreductases was detected in *C. divulgatum* with a FADox protein, FAD-dependent oxidoreductase of DAO superfamily (CSP5_1916, 2-fold increase). The cold-shock response in *P. furiosus* showed upregulation of biosynthesis of branched-chain amino acids and methionine. Our data revealed the upregulation of methionine, as well [33]. However, solute-binding proteins CipA and CipB identified as two major membrane glycoproteins in *P. furiosus*, representing archaeal type of bacterial cold shock protein (Csp) family, are non-existent in *C. divulgatum*.

### 3.5. Most Abundant Proteins

Combined, alpha and beta subunits of thermosome were the most significant proteins, making up to 3.18% and 3.29% of the total identified proteins in both, exponentially-grown and cold-imposed *C. divulgatum*, which, unlike *Sulfolobales* [44], does not encode in its genome a gamma-subunit to form α, β, γ heterooligomeric complex in response to the cold. Further, the most-abundant proteins (Table S2) were glutamate dehydrogenase (CSP5_1203, 2.0 and 2.19%) peroxiredoxin (CSP5_0886, 1% of the sum of identified proteins under both conditions), rubrerythrin (CSP5_0891, ca. 0.9% under both conditions), malate dehydrogenase (CSP5_0838, ca. 0.87% and 0.83%), and 2-oxoacid ferredoxin oxidoreductase (CSP5_0284, ca. 0.72% and 0.79%) and S-adenosylmethionine (SAM) synthetase (CSP5_0006, 0.8% and 0.97%, i.e., 20% increase in the cold). Glutamate dehydrogenases (GDH) are linked in humans to many cellular processes—including acid-base homeostasis, redox homeostasis, ammonia metabolism and lipid biosynthesis [45]. In bacteria and archaea, GDH are known to be important for carbon and nitrogen metabolism and responsible for oxidative deamination of glutamate and for reductive inclusion of ammonium into 2-oxoglutarate [46]. For *Thermococcus profundus* grown on peptides, GDH function was suggested for deamination of glutamate, accompanied with formation of NAD(P)H+(reduced) and 2-oxoglutarate, which is channelled into tricarboxylic acids cycle and used as an energy source [47]. We consider a similar role of GDH in deamination of glutamate originated from polypeptides used for cultivation of *C. divulgatum*. Other proteins associated with oxidative stress were also found synthesised at a high level, such as vitamin B_12_-dependent ribonucleotide reductase (CSP5_2009, ca. 0.48% under both conditions), and lactaldehyde dehydrogenase (CSP5_1304, ca. 0.51% under both conditions). The abundance of glutamate dehydrogenase, which tops the list of most-produced cellular proteins with an enzymatic function, does reflect the lifestyle of *Cuniculiplasma* as polypeptide-scavenging organism, and which enables the conversion of glutamate from externally acquired glutamate-containing proteinaceous substrates into α-ketoglutarate that is then funnelled into the tricarboxylic acid cycle. Interestingly, SAM synthetase is one of the most abundant proteins in the cold proteome (5th ranked, 0.97%), which provides the substrate for SAM-dependent methyltransferases. SAM synthetase generates the methyl group donor involved into polyamine synthesis, methylation of DNA and other organic molecules [48] that may contribute to the cold stress response in *C. divulgatum*. While the *Cuniculiplasma* genome encodes 12 SAM-dependent methyltransferase homologues (including one pseudogene), only three of them were detected in this proteome dataset: (i) CSP5_0484 (low-identity to isoaspartyl methylransferase from *Archaeoglobus* and 6% downregulated in the cold), (ii) CSP5_0705 (50% AA similarity with bacterial ubiquinone/menaquinone biosynthesis C-methyltransferases and arsenite methyltransferase in *Methanosarcina*, with exactly the same expression levels under both conditions) and (iii) CSP5_1828 (48% AA similarity with bacterial rRNA guanine or uracil methyltransferases, 4% up in the cold). Their expression levels (ranging from 0.1% to 0.15% fmol) did show less than 6% variations under both conditions. FKBP-type peptidyl-prolyl cis-trans isomerase was another abundant protein (CSP5_1482, 0.67% of total proteome, which increased its numbers in the cold (5% up)). These proteins, apart from PPIase activity, also demonstrate a chaperone-like activity [49], supporting their importance of protein folding under stress conditions.

Protein folding is a critical factor, and chaperones, which are well-known for being involved in the general stress response, exhibited a lower expression in the cold. DnaK (CSP5_1162) was 2.4% down, Hsp20 (CSP5_0928 and CSP5_1387)was 17% and 4% down, respectively, DnaJ (CSP5_1163) 30% down, and GrpE (CSP5_1161), 15% down), although they were expressed at very low levels (0.1–0.3% fmol) (Table S2). Iron-sulfur cluster assembly protein (CSP5_1945, ca. 0.125 vs. 0.131%) and peptide methionine sulfoxide reductase (CSP5_1232, 0.088 vs. 0.087%) were found to be expressed at significant low levels (ca. 0.125 vs. 0.131%); these are redox enzymes commonly over-synthesised under oxidative stress periods by acidophilic bacteria [50].

## 4. Conclusions

Under cold-shock conditions, the majority of the top 10 most-abundant proteins (0.73–2 fmol% of the quantifiable proteins in this dataset) of *C. divulgatum* S5^T^ were marginally, yet statistically reliably, overexpressed. These included proteins involved in folding (thermosome subunits and FKBP-type peptidyl-prolyl *cis-trans* isomerase), glutamate deamination (glutamate dehydrogenase, the most abundant protein with enzymatic function), methylation (SAM synthetase), citric acid cycle (malate dehydrogenase and 2-oxoacid ferredoxin oxidoreductase) and in signal transduction pathways. Growth-associated elongation factor 1-alpha (down 1.4%), rubrerythrin and all four peroxiredoxins present in this dataset (equal levels under both conditions) were the only exceptions.

Low-abundance proteins (0.001–0.005 fmol%) that showed the greatest fold-changes in the proteome of actively metabolising *C. divulgatum* cells were the enzymes mainly involved in proteolytic machinery. Stomatin—a regulator of protease activity, ATP-dependent LonB protease, alongside ABC transporters and a cytochrome C oxidase of the heme copper oxidase 1 superfamily—were shown to be among the most highly expressed proteins during active growth. Cold-shock proteome analysis has pinpointed proteins that exhibited overexpression from functional categories ‘Amino acid transport and metabolism’, ‘Energy production and conversion’, ‘Coenzyme transport and metabolism’ and proteins belonging to unknown function groups. Overall, this study provided first insights into the intracellular proteome of *C. divulgatum* and suggested several potential mechanisms underlying its physiological adaptation to a short-term cold shock: overexpression in pathways for aspartate, methionine and cysteine synthesis and also in central metabolic pathways for carbohydrates transformation, pyrimidine biosynthesis and coenzyme transport. Since *C. divulgatum* is a ubiquitous acidophilic microorganism, these adaptive mechanisms could contribute to their broad distribution across a variety of acidic environments with different temperatures.

## Data

Data are available via ProteomeXchange with identifier PXD017828.

## Supplementary Materials

Supplementary materials can be found at https://www.mdpi.com/2076-2607/8/5/759/s1.
